# Co-infection of dengue and COVID-19: A case report

**DOI:** 10.1371/journal.pntd.0008476

**Published:** 2020-08-03

**Authors:** Morgane Verduyn, Nathalie Allou, Virgile Gazaille, Michel Andre, Tannvir Desroche, Marie-Christine Jaffar, Nicolas Traversier, Cecile Levin, Marie Lagrange-Xelot, Marie-Pierre Moiton, Stella Hoang

**Affiliations:** 1 Department of Respiratory Medicine, University Hospital Centre of Réunion, Saint-Denis, France; 2 Department of Dermatology, University Hospital Centre of Réunion, Saint-Denis, France; 3 Department of Microbiology, University Hospital Centre of Réunion, Saint-Denis, France; 4 Department of Infectious Diseases, University Hospital Centre of Réunion, Saint-Pierre, France; London School of Hygiene & Tropical Medicine, UNITED KINGDOM

Dengue and coronavirus disease 2019 (COVID-19) may share clinical and laboratory features.

Reunion Island is a French overseas department located in the Indian Ocean with a population of more than 850,000 inhabitants. Due to its tropical climate, Reunion Island is at risk of arbovirus outbreaks. An increase in the number of dengue cases has been reported on the island since the beginning of 2018, with 3 different serotypes circulating mostly in austral summer. According to the last epidemiological report of March 30, 2020 from Santé Publique France, 3,144 new cases of dengue have been diagnosed since the beginning of 2020 in Reunion Island [1]. On March 2020, the first COVID-19 cases were imported to the island from metropolitan France by airplane.

We report the case of an 18-year-old male living in Reunion Island, with no relevant past medical history except occasional migraines. Our patient travelled back from Strasbourg (initial French epicenter of COVID-19) to Reunion Island on March 18, 2020. After his arrival, he returned to his parents’ home, respected national confinement guidelines, and only went shopping once.

The onset of symptoms occurred on April 3, with fever (39°C), asthenia, anorexia, and headache. On April 4, he tested positive in the emergency department for severe acute respiratory syndrome coronavirus 2 (SARS-CoV-2) infection by reverse transcription (RT)-PCR (E gene, RdRP gene, and N gene positive), the causative virus of COVID-19. He was discharged from the emergency room after diagnosis.

On April 5, an itchy erythema rash appeared. He came back to the hospital on April 7 for persistent fever (38.7°C), arthromyalgia, dyspnea with polypnea (respiratory rate of 24 breaths per minute), and itchy maculopapular rash. The dengue rapid test was positive (NS1 antigen+) in the emergency department. Therefore, he was hospitalized the same day in the COVID-19 unit of St Denis University Hospital Center.

**Fig 1:**
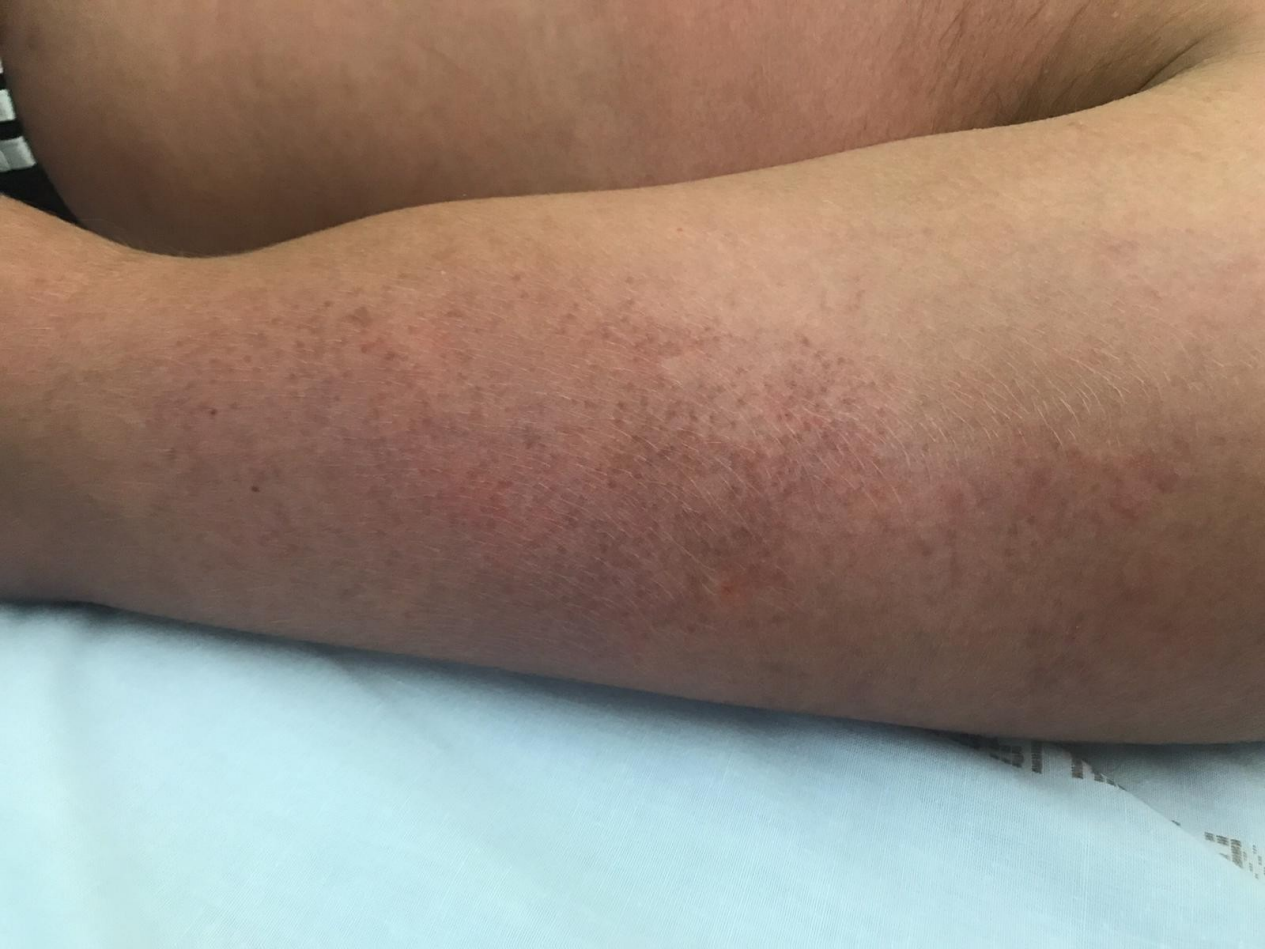
Photograph at hospital admission: r​oseoliform maculopapular exanthema with healthy skin intervals on left arm.

**Fig 2:**
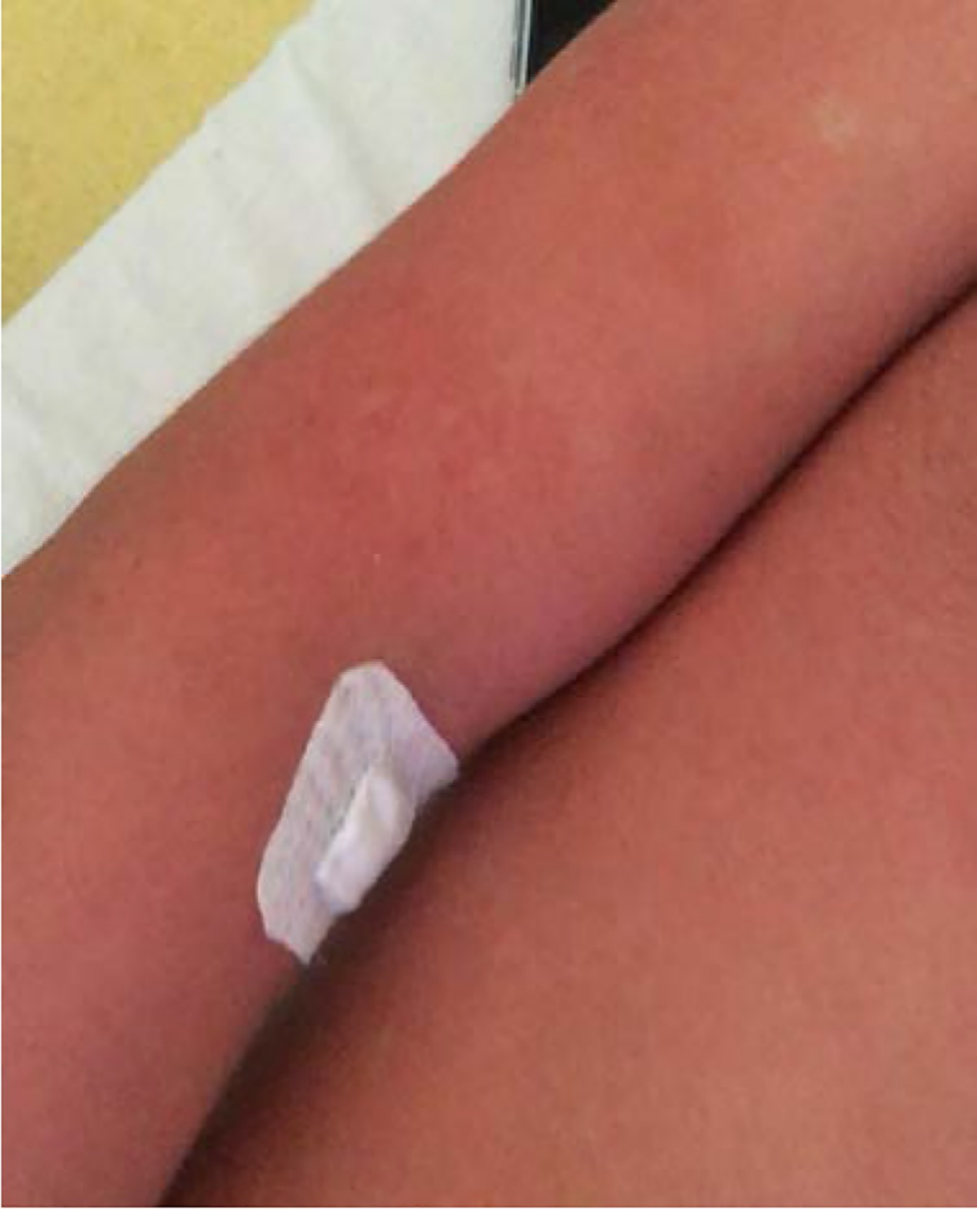
Photograph during hospitalization: diffuse exanthema with ​rounded island of sparing (“white island in a sea of red”)​.

**Fig 3:**
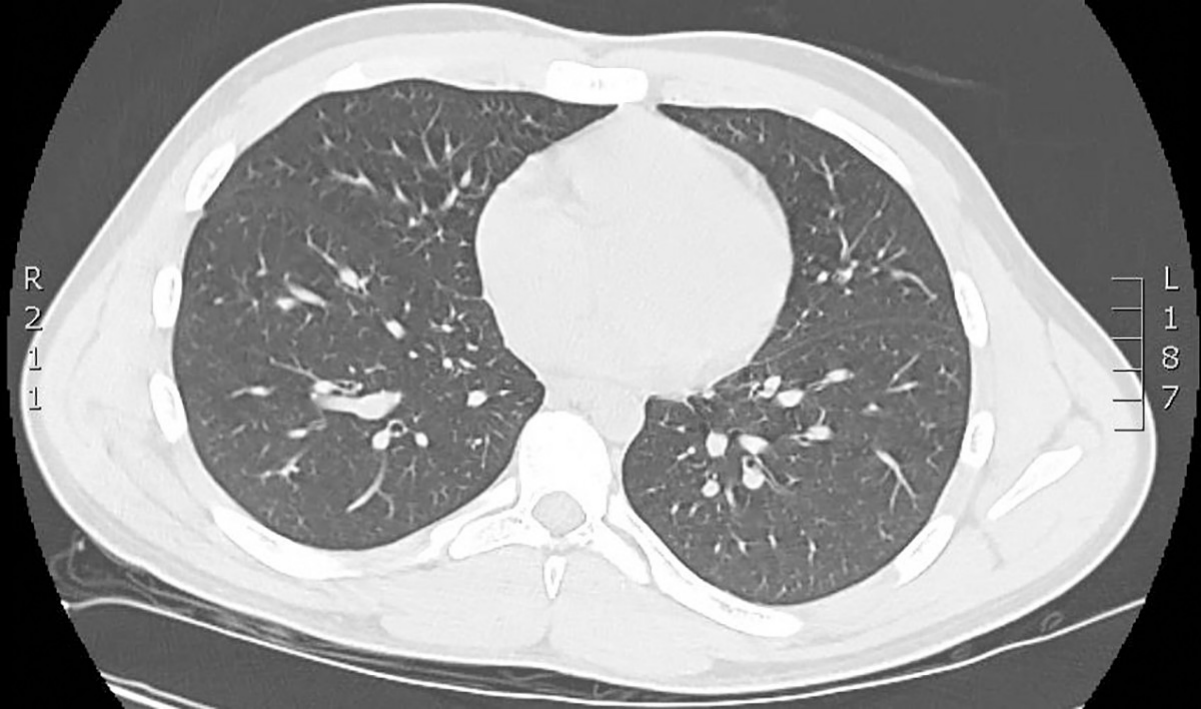
CT scan at admission was normal. CT, computed tomography.

The physical examination revealed a body temperature of 38°C, blood pressure of 112/63 mmHg, pulse of 63 beats per minute, and oxygen saturation of 99% in ambient air. He had dry cough (since February) and no chest pain. Pulmonary auscultation was normal. He had no hematuria. He described retro-orbital eye pain and mild photophobia, with anorexia, nausea, and vomiting. He had infracentimetric cervical lymphadenopathies. Skin examination showed a roseoliform maculopapular exanthema of the trunk, limbs, and face, which rapidly evolved into a scarlatiniform-like rash. There were no real intervals of healthy skin but rounded islands of sparing (“white islands in a sea of red”) (Figs 1 and 2). There was no mucosal involvement nor hand and feet affection involvement. The itching had stopped, and there was no scratching lesion. At admission, he had thrombocytopenia (platelet count 106 × 10^9^/mL), leucopenia (1.7 × 109/mL), lymphopenia (0.6 × 10^9^/mL), and neutropenia (1. × 10^9^/mL). Liver function test were subnormal (aspartate aminotransferase 51 U/L and alanine aminotransferase 23 U/L). C-reactive protein was normal (4.7 mg/L). Serotype 1 dengue was confirmed by RT-PCR and positive serology (immunoglobulin M [IgM]: 3.9 and IgG: 2.1) on day 6 after the onset of symptoms. The computed tomography (CT) scan performed at admission was normal without any ground glass opacities nor consolidations (Fig 3).

Fever above 39°C lasted 10 days, and the patient’s symptoms gradually improved. He returned home after 7 days of hospitalization. After initial worsening of thrombocytopenia (41 × 109/mL) and cytolysis (alanine aminotransferase: 545 U/L, aspartate aminotransferase: 621 U/L), the biological balance had started to improve at the end of hospitalization.

The patient’s parents tested negative for SARS-CoV-2 and declared a dengue at the same time.Informed consent was given orally by the individual to participate in this local retrospective observational study, which was approved by the local ethics committee and was declared to the Commission Nationale de l’Informatique et des Libertés (French Data Protection Agency or CNIL MR004). Informed consent was also obtained for publication of this case report with photographs.

To our knowledge, this is the first case of confirmed co-infection of dengue and COVID-19. In Singapore, 2 patients initially tested positive with a dengue rapid test. Ultimately, RT-PCR for dengue was negative, and both patients tested positive for SARS-CoV-2 infection by RT-PCR [2].

Distinction between dengue fever and COVID-19’s clinical features may be difficult. Our patient’s symptoms are consistent with dengue, including prolonged fever, facial flushing skin erythema, generalized body ache, myalgia, arthralgia, retro-orbital eye pain, photophobia, rubeoliform exanthema, and headache [3–4]. But some of them may be also consistent with clinical symptoms of COVID-19 [5]. Thrombocytopenia and elevated liver enzymes are also reported in both diseases. Thrombocytopenia and cytolysis were reported, respectively, in 36.2% and 21.3% of the patients with COVID-19 [5]. As in dengue fever [6], immune-mediated damage or direct cytotoxicity due to active viral replication in hepatic cells may be involved in hepatic injuries in COVID-19, but it could be also related to hypoxic hepatitis due to anoxia, reactivation of preexisting liver disease, or drug-induced liver injury (such as paracetamol, antiviral agents, etc.) [7].

In our case, making the hypothesis of a COVID-19 contamination during the flight on March 18 (where a confirmed COVID-19 passenger had been identified), an incubation period until symptoms on April 3 would have been longer than what has been described so far [8]. It is more likely that our patient was asymptomatic for SARS-CoV-2 infection but that RT-PCR was still positive on day 17, as previously described [9], and that most of his symptoms were related to dengue fever. In that case, it would have been interesting to use SARS-CoV-2 serology to identify a real and active co-infection from a case of dengue fever occurring in a SARS-CoV-2 cured patient.

Nonetheless, our patient presented a quite severe dengue infection with no previous episodes to his knowledge. Dengue serology on day 6 was positive for IgG (2.1) and IgM (3.9). One hypothesis could be that SARS-CoV-2 infection is more likely to give more severe symptoms in the case of co-infection.

Recently, skin damage has been described in COVID-19, but none of it seems to be specific to COVID-19. In Italy, 14 of 18 patients with cutaneous manifestations developed an erythematous rash, and 3 patients developed widespread urticarial [10]. The main region involved was trunk, and itching was low or absent. In Thailand, dermatologists also reported the case of a patient with an exanthema with fever initially diagnosed as dengue; finally, the patient was diagnosed for COVID-19 infection [11]. In April 2020, a French dermatologist reported the appearance of pseudo-frostbite of the extremities, sudden appearance of persistent redness, and sometimes painful, temporary, hive-like lesions [12]. In tropical areas where COVID-19 and arboviruses coexist, clinical distinction between different skin symptoms may be difficult. In our case, rounded islands of sparing “white islands in a sea of red” seem to be more specific of dengue virus [13].

We described here the first confirmed case of co-infection of dengue fever and COVID-19. In tropical areas where arboviruses and COVID-19 may coexist, clinical diagnosis is difficult, and patients should be tested for both viruses. Larger studies are needed to evaluate increased morbidity of these co-infections.
